# Relationships between motor and cognitive functions and subsequent post-stroke mood disorders revealed by machine learning analysis

**DOI:** 10.1038/s41598-020-76429-z

**Published:** 2020-11-11

**Authors:** Seiji Hama, Kazumasa Yoshimura, Akiko Yanagawa, Koji Shimonaga, Akira Furui, Zu Soh, Shinya Nishino, Harutoyo Hirano, Shigeto Yamawaki, Toshio Tsuji

**Affiliations:** 1 Department of Rehabilitation, Hibino Hospital, Hiroshima, 731-3164 Japan; 2 Department of Neurosurgery, Graduate School of Biomedical and Health Science, Hiroshima University, Hiroshima, 734-8551 Japan; 3 Graduate School of Engineering, Hiroshima University, Higashi-Hiroshima, 739-8527 Japan; 4 Graduate School of Advanced Science and Engineering, Hiroshima University, Higashi-Hiroshima, 739-8527 Japan; 5 College of Engineering, Academic Institute, Shizuoka University, Hamamatsu, 422-8529 Japan; 6 Center for Brain, Mind and KANSEI Sciences Research, Hiroshima University, Hiroshima, 734-8551 Japan

**Keywords:** Psychology, Neurological disorders, Data mining, Machine learning

## Abstract

Mood disorders (e.g. depression, apathy, and anxiety) are often observed in stroke patients, exhibiting a negative impact on functional recovery associated with various physical disorders and cognitive dysfunction. Consequently, post-stroke symptoms are complex and difficult to understand. In this study, we aimed to clarify the cross-sectional relationship between mood disorders and motor/cognitive functions in stroke patients. An artificial neural network architecture was devised to predict three types of mood disorders from 36 evaluation indices obtained from functional, physical, and cognitive tests on 274 patients. The relationship between mood disorders and motor/cognitive functions were comprehensively analysed by performing input dimensionality reduction for the neural network. The receiver operating characteristic curve from the prediction exhibited a moderate to high area under the curve above 0.85. Moreover, the input dimensionality reduction retrieved the evaluation indices that are more strongly related to mood disorders. The analysis results suggest a stress threshold hypothesis, in which stroke-induced lesions promote stress vulnerability and may trigger mood disorders.

## Introduction

Depression is a common neuropsychiatric symptom, affecting 18–78% of patients during the acute and subacute phase after a stroke, and been reported to negatively affect functional and cognitive recovery^1–5^. Apathy, defined as reduced motivation to engage in activities or the lack of motivation, is often observed after a stroke^2,6,7^. The diagnostic term ‘apathy’ does not appear in the Diagnostic and Statistical Manual of Mental Disorders, fourth edition (DSM-IV); only a few symptoms of ‘apathy’ appear as part of the diagnostic criteria for a major depressive episode; i.e. markedly diminished interest, suggesting apathy to be a part of depression in the psychiatric field^3^. Thus, we previously examined the relationships between post-stroke depression (PSD), functional recovery, and lesion location. Following the categorisation of PSD into two core symptom dimensions (namely, depressive and apathetic symptoms), we demonstrated that these dimensions may have different underlying neuroanatomic mechanisms and different effects on functional recovery^1,3,8^. These dimensions also appear to be associated with cognitive dysfunction, which may impair functional recovery after a stroke^3–5^. Anxiety is also a common mood disorder observed after a stroke, and is highly correlated with symptoms of depression^2,9,10^. Patients with comorbid anxiety and depression may exhibit greater impairment while performing activities of daily living (ADL) than those with depression or anxiety alone^2,10,11^.

Stroke survivors present notable but varying degrees of residual disability, either physical disability or cognitive disorder, which hinder their ability to perform ADL^1,12,13^. Moreover, residual physical disability causes distress and depression in many stroke patients^1,14,15^. This is traditionally thought to be a normal mourning process corresponding to a reactive psychological mechanism. However, when such mood disorders become severe and morbid, they indicate neuropsychiatric alterations, such as PSD, impeding patients to actively participate in rehabilitation and adversely affecting their functional recovery^3^. Thus, early diagnosis and intervention are crucial for PSD. However, PSD has been diagnosed using various scales as a measure to detect major depression, which is yet to be validated for the earlier detection of PSD, given that PSD is multifactorial, and associated neurological symptoms may hinder the detection process^16^. Hence, there is a need for a screening and diagnosis tool for PSD.

PSD has been reported to be associated with stroke severity and the degree of functional physical and cognitive impairment; however, it is uncertain whether the etiological mechanism of PSD is associated with the “reactive” psychological mechanism (mourning process against physical impairment) or other biological factors associated with brain damage^4,16^. One of the traditional hypotheses on the PSD mechanism was “threshold hypothesis”, the accumulation of lesions exceeding a threshold predispose to depression^5^, consistent with many previous reports demonstrating the association between the accumulation of lacunar infarcts within the basal ganglia, thalamus, and deep white matter and PSD^16^. Previous studies on the role of psychosocial stressors as risk factors for psychological illnesses (such as depression and anxiety) indicate that the impact of a stressful event is determined by the subject’s perception^17^. Thus, vascular lesions may result in the vulnerability to depression through the reduction of stress responses^5,17^. Stroke itself is a great psychological stress, and it is believed that depression can easily occur if a stroke renders patients vulnerable to stress.

After a stroke, various physical disorders, cognitive dysfunction, and mood disorders associated with stress responses are intricately intertwined, making it difficult to understand the aetiology of PSD and, therefore, making the diagnosis of PSD challenging. Therefore, it is necessary to clarify the relevance of such complicated post-stroke symptoms to improve rehabilitation outcomes. In this paper, we focus on mood disorders associated with the vulnerability to stress, namely, depression, apathy, and anxiety, to clarify their cross-sectional relationship with motor/cognitive function after a stroke. We comprehensively analysed this relationship using a machine learning approach to unveil the pathogenesis of post-stroke mood disorders.

## Materials and methods

### Participants

We used clinical data obtained from 274 stroke inpatients (age: 64.9 ± 10.7 years) at the Hibino Hospital, who could perform psychological and cognitive function tests. All patients provided informed consent. They were admitted to the Kaifukuki Rehabilitation Ward, where the inpatients were hospitalised (admitted) within two months of onset after acute treatment for stroke; rehabilitation was performed for the inpatients for up to 180 days and up to 3 hours a day. The patients under treatment of major psychiatric illnesses, such as major depression, bipolar disorder, schizophrenia, or schizoaffective disorder, were excluded (in this study, one patient had a history of autonomic imbalance, and one had a history of insomnia/neurosis, but underwent the treatment and was treatment-free on admission). The type of stroke was haemorrhage or occlusive stroke (infarction and transient ischaemic attack; TIA). Infarction in one patient was associated with mild subarachnoid haemorrhage. The study was approved by the Ethics Review Committee of the Hiroshima University Epidemiological Research and the Ethics Review Committee of the Shinaikai Hibino Hospital, and was performed in accordance with relevant guidelines and regulations.

### Assessment of cognitive function

Cognitive function was examined using the Mini-Mental State Examination with scores ranging from 0 to 30 and the Trail Making Test. Attention deficit was systematically evaluated using the Clinical Assessment of Attention Deficit, as described previously^18^ along with another Trail Making Test. Spatial neglect was examined using the Behavioural Inattention Test, and memory was examined using the Rivermead Behavioural Memory Test. The tasks analysed to assess cognitive function are listed in Table 1.

### Measurements of stroke severity

The Functional Independence Measure (FIM) version 3.0 contains 18 items (13 motor and 5 cognitive items) that comprise an observer-rated summed rating scale for evaluating disability in terms of dependency (the lower the score, the greater the disability). The FIM is widely used to quantify disability in stroke patients^19^. Hence, all patients were examined for disability using the FIM within a week after admission and at discharge. The FIM improvement rate was calculated as follows: $[(\text {FIM score on discharge}) - (\text {FIM score on admission})]/[\text {period of hospitalisation (weeks)}]$.

Motor impairment in hemiplegic stroke patients was measured using the Brunnstrom Recovery Scale (BRS), wherein movement patterns were evaluated in the upper limb, fingers, and lower limb, and motor function was evaluated according to the stages of motor recovery^19^. The scale defines recovery only in broad categories, which correlate with those of progressive functional recovery (the lower the score, the greater the disability). The following analysis was performed by summing the stages of BRS of the upper limb, fingers, and lower limb.

The presence or absence of ataxia and aphasia was evaluated at admission.

Lesion location of infarction was assessed using magnetic resonance imaging (MRI) and that of haemorrhage was assessed using MRI or computed tomography (CT), and categorised into brainstem, cerebellum, right or left basal ganglia, right or left subcortical, and right or left cortical.

### Psychological assessment

The Hospital Anxiety and Depression Scale (HADS) was used to identify depression and anxiety, and the apathy score was used to identify apathy. We derived HADS-Depression and HADS-Anxiety scores using the HADS, and patients with HADS-Depression and HADS-Anxiety scores above 9 were classified as having PSD and anxiety, respectively. In addition, patients were adjudged to have apathy when they had an apathy score above 16. To assess stress, we used the Japanese version^20^ of the perceived stress scale (JPSS) originally developed by Cohen et al.^21^. This scale is widely used to measure the degree to which situations in a subject’s life are appraised as stressful.

### Proposed machine learning approach

**Figure 1:**
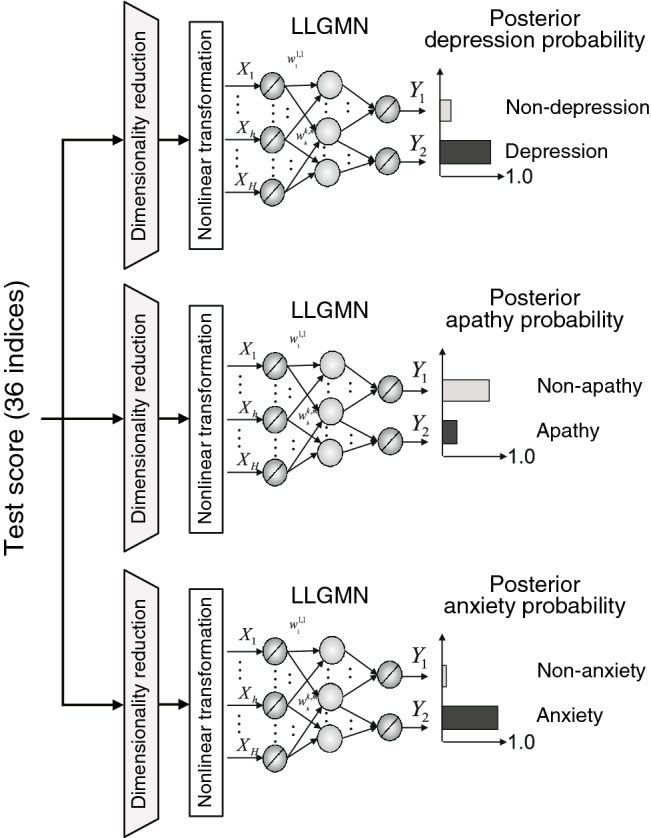
Overview of discriminant machine learning model of mood and functional disorders.

To analyse the relationship between mood disorders and motor/cognitive functions, we used a probabilistic artificial neural network called log-linearized Gaussian mixture network (LLGMN)^22^. This network enables the estimation of the statistical distribution of sample data based on machine learning and the prediction of the posterior probability of the class for unknown input data. We propose a mood disorder identification model composed of three LLGMNs, as illustrated in Fig. 1. We independently predicted the posterior probabilities of each mood disorder, namely, PSD, apathy, and anxiety. The input to each LLGMN is a *P*-dimensional evaluation index, ${\mathbf {z}}^{(n)} = [{z}^{(n)}_1,{z}^{(n)}_2,\ldots ,{z}^{(n)}_P]^T\in \mathbb {R}^P$, obtained from the eight abovementioned evaluation tests, where *n* identifies the patient. The output is a two-dimensional posterior probability vector, ${\mathbf {Y}}^{(n)}_r\in \mathbb {R}^2$, representing the absence or presence of a mood disorder, with $r = 1,2,3$ indicating PSD, apathy, and anxiety, respectively.

We first divided the 274 patients into four groups: control, depression, apathy, and anxiety groups (Table 1). The machine learning analysis was conducted for each combination of the control group and mood disorder groups. The training dataset comprised evaluation indices ${\mathbf {z}}^{(n)}$ ($n=1,2,\ldots ,N$) of *N* patients from each combination as training inputs, and the corresponding labels (absence/presence of mood disorders) ${\mathbf {Q}}^{(n)}_r \in \mathbb {R}^2$. The proposed model was trained using error backpropagation, and the prediction accuracy was verified using the validation dataset composed of the data excluded from the training dataset. Posterior probabilities ${\mathbf {Y}}^{(n^{\prime })}_r$ of each mood disorder were predicted by inputting validation inputs ${\mathbf {z}}^{(n^{\prime })}$ ($n^{\prime }=1,2, \ldots ,N^{\prime }$) to the model. The evaluation accuracy was then evaluated using the area under the curve (AUC) from the receiver operating characteristic (ROC) curve on the predicted posterior probability ${\mathbf {Y}}^{(n^{\prime })}_r$ and true ${\mathbf {Q}}^{(n)}_r$.

**Table 1:** Clinical characteristics of subjects categorized into psychiatric group in this study.

	Control group $(n \quad = 80)$	Depression group $(n \quad = 40)$	Apathy group $(n \quad = 80)$	Anxiety group $(n \quad = 40)$	*p*-value	*p*-value of post-hoc test
Age (years)	65.9 ± 10.0	61.7 ± 11.3	64.4 ± 11.7	65.7 ± 9.8	0.2094	
Sex (male), *n* (%)	67 (83.8%)	32 (80.0%)	65 (81.3%)	34 (85.0%)	0.9143	
Past history of stroke, *n* (%)	10 (12.5%)	5 (12.5%)	15 (18.8%)	8 (20.0%)	0.564	
**Disease**						
Infarction, *n* (%)	58 (72.5%)	30 (75.0%)	63 (78.8%)	34 (85.0%)	0.2932	
Hemorrhage, *n* (%)	19 (23.8%)	10 (25.0%)	16 (20.0%)	5 (12.5%)		
TIA, *n* (%)	3 (3.8%)	0 (0.0%)	1 (1.25%)	0 (0%)		
**Lesion location**						
Rt basal ganglia, *n* (%)	19 (23.8%)	6 (15.0%)	16 (20%)	5 (12.5%)	0.4394	
Rt subcortical, *n* (%)	25 (31.3%)	12 (30.0%)	27 (33.8%)	13 (32.5%)	0.976	
Rt cortical, *n* (%)	14 (17.5%)	6 (15.0%)	10 (12.5%)	5 (12.5%)	0.8095	
Lt basal ganglia, *n* (%)	19 (23.8%)	17 (42.5%)	18 (22.5%)	13 (32.5%)	0.0907	
Lt subcortical, *n* (%)	25 (31.3%)	17 (42.5%)	29 (36.3%)	18 (45.0%)	0.4307	
Lt cortical, *n* (%)	12 (15.0%)	6 (15.0%)	13 (16.3%)	5 (12.5%)	0.9614	
Cerebellum, *n* (%)	3 (3.8%)	3 (7.5%)	7 (8.8%)	2 (5.0%)	0.5909	
Brainstem, *n* (%)	10 (12.5%)	6 (15.0%)	14 (17.5%)	7 (17.5%)	0.8199	
**Stroke severity**						
Motor FIM on admission	62.8 ± 20.8	62.0 ± 22.5	63.7 ± 20.6	66.9 ± 22.0	0.6056	
Cognitive FIM on admission	29.2 ± 5.5	25.3 ± 7.2	26.3 ± 7.3	26.4 ± 8.4	**0.0144**	C. vs D. 0.0131
Motor FIM at discharge	82.5 ± 10.1	80.2 ± 10.1	81.0 ± 9.8	81.7 ± 8.5	0.4615	
Cognitive FIM at discharge	32.2 ± 3.7	29.5 ± 5.0	29.9 ± 4.7	30.4 ± 5.3	**0.0002**	C. vs D. 0.0025, vs Ap. 0.0006
Motor FIM improvement rate	3.3 ± 3.5	1.9 ± 1.8	2.2 ± 2.2	1.8 ± 2.7	0.0779	
Cognitive FIM improvement rate	0.6 ± 1.4	0.4 ± 0.5	0.4 ± 0.5	0.4 ± 0.7	0.6579	
**Physical disability**						
Paresis						
BRS total score on admission	15.3 ± 3.9	14.3 ± 4.4	14.8 ± 4.3	14.7 ± 4.4	0.5623	
BRS total score at discharge	16.4 ± 3.2	15.9 ± 3.0	16.3 ± 2.8	16.2 ± 2.8	0.2393	
Ataxia, *n* (%)	4 (5.0%)	2 (5.0%)	9 (11.3%)	3 (7.5%)	0.4511	
Aphasia, *n* (%)	8 (10.0%)	8 (20.0%)	11 (13.8%)	6 (15.0%)	0.5192	
Period from onset to examination (days)	20.8 ± 28.3	25.9 ± 25.0	20.2 ± 19.9	20.1 ± 22.0	0.2999	
Hospitalization period (days)	49.7 ± 45.4	67.0 ± 47.5	59.1 ± 47.0	60.7 ± 48.0	0.1071	
**Psychological assessment**						
HADS-Depression	2.3 ± 1.7	11.1 ± 2.4	6.7 ± 3.4	7.4 ± 3.8		
HADS-Anxiety	2.9 ± 2.0	7.9 ± 4.1	6.4 ± 3.3	10.7 ± 2.3		
Apathy score	5.1 ± 2.8	16.7 ± 6.9	20.2 ± 4.2	14.5 ± 6.5		
JPSS	16.1 ± 6.5	25.7 ± 6.6	23.3 ± 6.4	25.2 ± 6.9	$$< \mathbf{0.0001} $$	C. vs D., vs Ap., vs Anx. $$< 0.0001 $$
**Cognitive function test**						
MMSE	27.5 ± 2.4	26.1 ± 3.9	26.2 ± 4.0	26.9 ± 4.0	0.1122	
BIT conventional subtest	141.4 ± 8.0	137.9 ± 10.3	138.2 ± 11.0	140.0 ± 7.3	**0.0051**	C. vs D. 0.0254, vs Ap. 0.0079
BIT behavioural subtest	78.5 ± 6.9	76.5 ± 6.1	75.7 ± 11.4	77.7 ± 6.0	**0.0038**	C. vs D. 0.0136, vs Ap. 0.0208
Digit span forward	5.5 ± 1.1	5.3 ± 1.3	5.4 ± 1.0	5.6 ± 1.4	0.335	
Digit span backward	4.1 ± 1.0	3.8 ± 1.3	3.9 ± 1.2	4.3 ± 1.3	0.3308	
Tapping span forward	5.7 ± 1.4	5.5 ± 1.2	5.4 ± 1.3	5.7 ± 1.2	0.374	
Tapping span backward	4.9 ± 1.4	4.8 ± 1.3	4.4 ± 1.2	4.9 ± 1.4	0.1417	
Visual cancellation Kana accuracy	93.8 ± 9.9	90.8 ± 11.4	93.1 ± 8.4	93.8 ± 7.2	0.595	
Visual cancellation $\Delta$ accuracy	97.1 ± 6.9	96.8 ± 5.9	97.1 ± 4.2	98.1 ± 3.4	0.6413	
Visual cancellation * accuracy	97.7 ± 9.7	97.3 ± 7.2	97.5 ± 5.9	98.7 ± 3.4	0.2696	
Visual cancellation 3 accuracy	98.0 ± 9.1	97.4 ± 6.0	97.6 ± 6.1	97.8 ± 5.0	0.1724	
Visual cancellation Kana time	136.8 ± 42.4	183.1 ± 126.5	175.2 ± 100.5	169.0 ± 59.5	**0.0007**	C. vs D. 0.0148, vs Ap. 0.0024, vs Anx. 0.0179
Visual cancellation $\Delta$ time	62.6 ± 22.9	78.0 ± 47.9	77.2 ± 38.3	78.2 ± 32.3	**0.0017**	C. vs Ap. 0.0036, vs Anx. 0.0170
Visual cancellation * time	74.9 ± 25.5	102.1 ± 94.2	102.2 ± 86.9	91.2 ± 33.8	**0.0013**	C. vs D. 0.0169, vs Ap. 0.0039, vs Anx. 0.0320
Visual cancellation 3 time	113.0 ± 31.8	140.7 ± 60.4	137.9 ± 64.1	132.1 ± 41.6	**0.0037**	C. vs D. 0.0190, vs Ap. 0.0203
SDMT number of wrong answers	0.8 ± 1.6	0.9 ± 1.0	1.0 ± 1.3	1.1 ± 1.4	0.19	
SDMT achievement rate	35.9 ± 11.7	28.2 ± 11.0	28.2 ± 12.0	29.4 ± 11.5	$< \mathbf{0.0001}$	C. vs D. 0.0047, vs Ap. 0.0002, vs Anx. 0.0257
Memory updating 3 span accuracy	64.7 ± 22.4	62.8 ± 23.1	59.1 ± 24.6	60.1 ± 25.5	0.5064	
PASAT: 2-second accuracy	47.2 ± 22.5	34.4 ± 19.6	37.2 ± 20.2	38.1 ± 20.2	**0.0038**	C. vs D. 0.0102, vs Ap. 0.0184
Position Stroop accuracy	96.0 ± 12.5	90.7 ± 18.9	93.7 ± 12.6	93.1 ± 16.3	**0.0095**	C. vs D. 0.0216
Position Stroop time	107.3 ± 43.4	138.3 ± 91.8	142.1 ± 81.9	118.6 ± 53.2	**0.0139**	C. vs Ap. 0.0120
CPT-SRT time	363.7 ± 99.9	410.5 ± 108.1	401.3 ± 91.4	386.1 ± 91.8	**0.0141**	C. vs Ap. 0.0194
CPT-X time	534.2 ± 87.6	563.2 ± 82.2	574.3 ± 103.1	555.2 ± 105.6	**0.0338**	C. vs Ap. 0.0355
CPT-AX time	547.3 ± 104.2	581.1 ± 133.0	589.5 ± 118.7	563.2 ± 119.4	**0.0413**	C. vs Ap. 0.0495
TMT part A time	55.0 ± 29.4	76.9 ± 51.6	70.9 ± 44.1	72.6 ± 45.7	**0.006**	C. vs D. 0.0357, vs Ap. 0.0195
TMT part B time	108.2 ± 38.6	146.7 ± 57.9	134.5 ± 58.9	146.1 ± 57.9	**0.0063**	C. vs D. 0.0302, vs Anx. 0.0265
Fail of TMT part A, *n* (%)	3 (3.8%)	1 (2.5%)	5 (6.3%)	1 (2.5%)	0.6961	
Fail of TMT part B, *n* (%)	22 (27.5%)	17 (42.5%)	36 (45.0%)	15 (37.5%)	0.1182	
RBMT profile	20.1 ± 3.5	17.8 ± 4.5	17.0 ± 4.7	17.7 ± 4.6	$< \mathbf{0.0001}$	C. vs D. 0.0105, vs Ap. $< 0.0001$, vs Anx. 0.0106

Differences in control and psychiatric grouping (depression, apathy and anxiety). All results are presented as mean ± S.D. or number (%). *p*-value was indicated using the $\chi ^{{2}}$ test for categorial values and Kruskal–Wallis analysis for continuous values. The post-hoc tests were done using the Steel–Dwass test. Significant *p*-values (< 0.05) are in bold.

*TIA* Transient Ischemic Attack, *FIM* Functional Independence Measure, *BRS* Brunnstrom Recovery Scale, *BIT* Behavioural Inattention Test, *MMSE* Mini-Mental State Examination, *HADS* Hospital Anxiety and Depression Scale, *JPSS* Japanese Perceived Stress Scale, *SDMT* Symbol Digit Modalities Test, *PASAT* Paced Auditory Serial Addition Test, *CPT* Continuous Performance Test, *SRT* Simple Reaction Time, *TMT* Trail Making Test, *RBMT* Rivermead Behavioural Memory Test, *C.* Control group, *D.* Depression group, *Ap.* Apathy group, *Anx.* Anxiety group.

### Input dimensionality reduction using partial Kullback–Leibler information

Estimating the statistical distribution of a high-dimensional input space $({\mathbf {z}}(n) \in \mathbb {R}^P)$ may lead to suboptimal solutions reflecting local minima. In addition, it is not possible to clarify the relationship between each evaluation index and mood disorder simply by predicting the absence or presence of the mood disorder using the LLGMN. Therefore, we reduced the input dimension using the partial Kullback–Leibler (KL) information measure^23^ and identified the most relevant indices related to each mood disorder.

The partial KL information measure is defined as

1 $$\begin{aligned} E_{r,\left[ {\mathbf {I}}+\bar{i}\right] } = \frac{I_r\left( {\mathbf {Q}}_r, \mathbf {Y}_{r,\left[ {\mathbf {I}}\right] }\right) }{I_r\left( {\mathbf {Q}}_r, \mathbf {Y}_{r,\left[ {\mathbf {I}}+\bar{i}\right] }\right) } = \frac{\sum _{n^{\prime }}^{N^{\prime }}I_r \left( {\mathbf {Q}}^{\left( n^{\prime }\right) }_r,{\mathbf {Y}}^{\left( n^{\prime }\right) }_{r,\left[ {\mathbf {I}}\right] }\right) }{\sum _{n^{\prime }}^{N^{\prime }}I_r \left( {\mathbf {Q}}^{\left( n^{\prime }\right) }_r,{\mathbf {Y}}^{\left( n^{\prime }\right) }_{r,\left[ {\mathbf {I}}+\bar{i}\right] }\right) }, \end{aligned}$$

where ${\mathbf {I}}\in {\mathbb {R}}^D$ is a dimension set reduced from the evaluation index vector ${\mathbf {z}}^{(n^\prime )}$, $\bar{i}$ is the reduction target dimension, ${\mathbf {Y}}^{(n^{\prime })}_{r,[{\mathbf {I}}]}$ and ${\mathbf {Y}}^{(n^{\prime })}_{r,[{\mathbf {I}}+\bar{i}]}$ are vectors representing the posterior probability distributions of the classes predicted by inputting the evaluation index vector with these dimensions reduced, and $I_r({\mathbf {Q}},{\mathbf {Y}})$ is the KL information between arbitrary probability distributions ${\mathbf {Q}}$ and ${\mathbf {Y}}$. The input dimensionality reduction proceeds as follows.

1. The number of reduced dimensions is initialised as $d=0$ ($D=P$), and the reduction dimensions is set as an empty set (${\mathbf {I}}$=$\phi$).
2. The evaluation index vector ${\mathbf {z}}^{(n^\prime )}_{[{\mathbf {I}}]} \in \mathbb {R}^D$ with the dimension set of ${\mathbf {I}}$ reduced is inputted to the LLGMN, and the partial KL information measure $I_r({\mathbf {Q}}^{(n^{\prime })}_r,{\mathbf {Y}}^{(n^{\prime })}_{r,[{\mathbf {I}}]})$ and AUC value ${\mathbf {A}}^{(n^{\prime })}_{r,[{\mathbf {I}}]}$ are calculated.
3. Let ${\bar{\mathbf {I}}} \in \mathbb {R}^D$ be the set of the remainder dimensions that have not been reduced from the evaluation index vector. In addition, let $\bar{i}\in {\bar{\mathbf {I}}}$ be an element of the set of the remainder dimensions. Then, ${\mathbf {I}}+\bar{i}$ represents the union in which the remainder dimension $\bar{i}$ is added to the reduced dimension set ${\mathbf {I}}$. The evaluation index vector ${\mathbf {z}}^{(n^\prime )}_{[{\mathbf {I}}+\bar{i}]} \in \mathbb {R}^{D-1}$ from which ${\mathbf {I}}+\bar{i}$ has been deleted is inputted to the LLGMN. The KL information measure $I_r({{\mathbf{Q}}}^{(n^{\prime })}_r,{{\mathbf{Y}}}^{(n^{\prime })}_{[{\mathbf {I}}+\bar{i}]} )$ is then calculated from the predicted posterior probability, ${\mathbf{Y}}^{(n^{\prime })}_{[{\mathbf {I}}+\bar{i}]}$.
4. The dimension maximising the partial KL information $\bar{i}_{\max }={\mathrm{arg}}\,{\mathrm{max}}_{\bar{i}\in {\bar{\mathbf {I}}}}$ $E_{r,[\mathbf {I}+\bar{i}]}$ is obtained using Eq. (1), and this dimension is added to $\mathbf {I}$ as a new reduction dimension.
5. After setting $d+1$ as a new reduced dimension *d*, steps 2 to 4 are repeated until $d = P-1$.

Following the above procedure, the model with the largest AUC is adopted for prediction.

### Relationship between evaluation indices and mood disorders

The proposed machine learning approach based on the LLGMN was evaluated using the dataset obtained from the 274 patients. The dataset was composed of the 36-dimensional evaluation index vector containing the results of the evaluation tests and the corresponding absence/presence of the mood disorder determined by the HADS and apathy scores. The input dimensionality reduction using the partial KL information enabled the extraction or representative indices for predicting PSD, apathy, and anxiety. Then, the ROC curve was obtained from the posterior probability of each mood disorder predicted by the LLGMN and labels (absence/presence of mood disorder). The prediction accuracy of mood disorders was evaluated using the AUC obtained by ten-fold cross-validation.

We compared the prediction accuracy of the proposed model with the reduced input dimension against three classification models: stepwise multiple linear regression, logistic regression, and partial least squares (PLS) regression. In the stepwise multiple linear regression, variables were selected using a forward-backward stepwise selection method. All variables were used in the logistic regression and PLS regression; the number of latent factors in the PLS regression was set to 3. The sensitivity, specificity, positive predictive value (PPV), and negative predictive value (NPV) that provided the maximum AUC were also calculated and compared with those of the proposed method. In this experiment, the positive (presence of mood disorder) and negative (absence of mood disorder) data included in the dataset were balanced throughout the analyses to eliminate the bias due to the mood disorder incidence.

Finally, we analysed and compared the decrease in AUC when one input dimension was disregarded from the input indices after dimensionality reduction using the partial KL information. This analysis enabled us to rate the importance of each evaluation index for the considered mood disorders.

### Statistical analysis

The differences in control and psychiatric grouping (depression, apathy, and anxiety) were assessed using the $\chi ^2$ test for categorial values and the Kruskal–Wallis analysis for continuous values. A post-hoc test was conducted based on the Steel–Dwass test. Values were considered to be significant at $p < 0.05$. JMP Pro 14.2.0 (SAS Institute Inc., Cary, NC, USA) was used for the analyses. To compare the AUC of the proposed method with those of other methods, a pairwise comparison with the proposed method was performed using the DeLong test^24^ with Holm adjustment. The DeLong test is a statistical test method for comparing two AUCs and is widely used owing to its non-parametric approach.

## Results

### Baseline structures

Table 1 presents the baseline data for stroke patients categorised into control, depression, apathy, and anxiety groups. Cognitive FIM was significantly lower in the mood disorder group. Age, the hospitalisation period, and physical disabilities (paralysis, ataxia, aphasia) were not significantly different in each group. In addition, in the presence of a mood disorder, JPSS was high and several cognitive functions were impaired.

### ROC analysis

**Figure 2:**
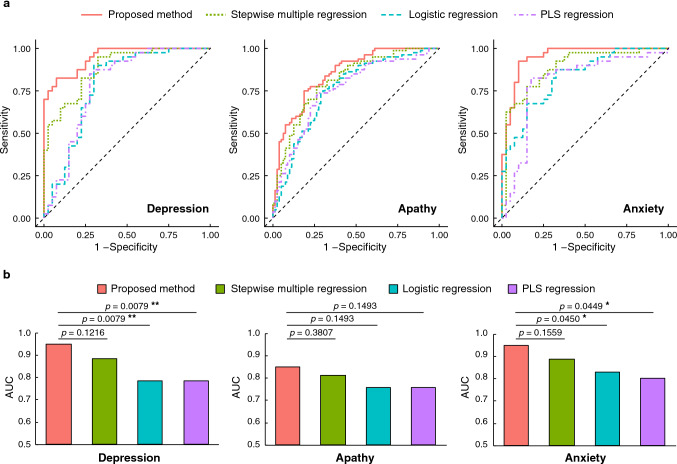
Results of ROC analysis obtained by ten-fold cross-validation. The red, green, blue, and purple lines/bars represent the proposed method, stepwise multiple regression, logistic regression, and partial least squares (PLS) regression, respectively. (**a**) ROC curve for each model. (**b**) AUC value for each model. Statistical test results obtained using the DeLong test for two correlated ROC curves with the Holm adjustment are also shown (* $p < 0.05$; ** $p < 0.01$).

The results of ROC analysis obtained from the comparison of the proposed model with three linear classification models are depicted in Fig. 2. The ROC curves of each method are overwrapped for each group (Fig. 2a). The proposed model with reduced input dimensionality revealed an AUC above 0.85 for all mood disorders, indicating its suitable classification accuracy, which reaches an AUC above 0.90 for PSD and anxiety (Fig. 2b). Overall, the AUC of the proposed model with reduced input dimensionality was the highest for all mood disorders among the evaluated models. The evaluation measures for each method are presented in Table 2.

**Table 2:** Evaluation measures providing the maximum area under ROC.

Group	Method	Measures			
		Sensitivity	Specificity	PPV	NPV
Depression	Proposed method	0.8250	0.9250	0.9167	0.8409
	Stepwise multiple linear regression	0.9500	0.6750	0.7451	0.9310
	Logistic regression	0.9000	0.7000	0.7500	0.8750
	PLS regression	0.8500	0.7250	0.7556	0.8286
Apathy	Proposed method	0.7500	0.8125	0.8000	0.7647
	Stepwise multiple linear regression	0.7750	0.7375	0.7470	0.7662
	Logistic regression	0.7375	0.7125	0.7195	0.7308
	PLS regression	0.7375	0.7250	0.7284	0.7342
Anxiety	Proposed method	0.9250	0.9000	0.9024	0.9231
	Stepwise multiple linear regression	0.7750	0.8500	0.8378	0.7907
	Logistic regression	0.8750	0.6750	0.7292	0.8438
	PLS regression	0.8250	0.8250	0.8250	0.8250

*PLS* Partial Least Squares, *PPV* Positive Predictive Value, *NPV* Negative Predictive Value.

We removed the indices one by one to evaluate the effect of the missing index on the classification accuracy of the proposed LLGMN model. Specifically, a removed index retrieving a large drop in accuracy would indicate a high contribution to mood disorder identification. The results from this evaluation are presented in Tables 3–5. The number of input indices after input dimensionality reduction was 11 for PSD, 14 for apathy, and 9 for anxiety. Consider Fig. 2 showing the AUC for PSD (0.949), apathy (0.850), and anxiety (0.950). For PSD, removing the JPSS, wrong answers in SDMT, and digit span backward results reduced the AUC by 20.1%, 9.82%, and 8.17%, respectively. For apathy, removing the JPSS, digit span backward, and tapping span backward results reduced the AUC by 15.0%, 6.97%, and 4.74%, respectively. For anxiety, removing the JPSS, digit span backward, and motor FIM on admission results reduced the AUC by 20.5%, 13.5%, and 10.3%, respectively.

**Table 3:** Indices contributing to PSD presented in descending order according to the reduction in AUC caused by their removal from the proposed machine learning approach.

Rank	Removed index	AUC reduction (%)
1	JPSS	20.1
2	SDMT wrong answers	9.82
3	Digit span backward	8.17
4	Memory updating 3-span accuracy	4.35
5	PASAT 2-second accuracy	4.28
6	CPT-SRT	3.69
7	TMT part B time	2.50
8	RBMT profile	2.50
9	Visual cancellation Kana time	1.91
10	Position Stroop time	0.791
11	Tapping span forward	0.198

*JPSS* Japanese Perceived Stress Scale, *PASAT* Paced Auditory Serial Addition Test, *CPT* Continuous Performance Test, *SRT* Simple Reaction Time, *TMT* Trail Making Test, *RBMT* Rivermead Behavioural Memory Test.

**Table 4:** Indices contributing to apathy presented in descending order according to the reduction in AUC caused by their removal from the proposed machine learning approach.

Rank	Removed index	AUC reduction (%)
1	JPSS	15.0
2	Digit span backward	6.97
3	Tapping span backward	4.74
4	Visual cancellation Kana time	4.14
5	CPT-SRT	3.44
6	Tapping span forward	3.27
7	Visual cancellation Kana accuracy	2.62
8	Cognitive FIM on admission	1.88
9	Visual cancellation $\bigtriangleup$ accuracy	1.78
10	BIT behavioural subtest	1.77
11	Digit span forward	1.75
12	Position Stroop time	1.34
13	SDMT wrong answers	0.90
14	Visual cancellation $\times$ time	0.129

*JPSS* Japanese Perceived Stress Scale, *CPT* Continuous Performance Test, *SRT* Simple Reaction Time, *FIM* Functional Independence Measure, *BIT* Behavioural Inattention Test, *SDMT* Symbol Digit Modalities Test.

**Table 5:** Indices contributing to anxiety presented in descending order according to the reduction in AUC caused by their removal from the proposed machine learning approach.

Rank	Removed index	AUC reduction (%)
1	JPSS	20.5
2	Digit span backward	13.5
3	Motor FIM on admission	10.3
4	Cognitive FIM improvement rate	6.91
5	TMT part B time	5.46
6	Position Stroop accuracy	4.14
7	Visual cancellation $\bigtriangleup$time	1.84
8	CPT-SRT	1.25
9	Digit span forward	0.263

*JPSS* Japanese Perceived Stress Scale, *FIM* Functional Independence Measure, *TMT* Trail Making Test, *CPT* Continuous Performance Test, *SRT* Simple Reaction Time.

## Discussion

We devised a machine learning approach to analyse the relationship between post-stroke mood disorders and indices obtained from functional evaluation tests. We confirmed that the proposed model could predict post-stroke neuropsychiatric symptoms (i.e. PSD and anxiety) with moderate to high accuracy, with an AUC above 0.85 for all the evaluated mood disorders (see Fig. 2). The classification characteristics of each method are summarised in Table 2, indicating that the proposed method can classify both negative and positive data with a relatively good balance. Therefore, the proposed non-linear model effectively predicts post-stroke neuropsychiatric symptoms and outperforms traditional linear classification.

PSD is widely thought to be associated with stroke severity and the degree of physical and cognitive impairment. In Table 1, the many cognitive function tests can be seen to be lower in depression, apathy, and anxiety groups than in control group. In addition, considering the severity after stroke, cognitive FIM was lower in the presence of a mood disorder at the time of admission and discharge. Cerebrovascular lesions, which are associated with depression or cognitive impairment through related mechanisms, result in poor prognosis for PSD patients^3–5,25^. It is believed that the presence of PSD interferes with ADL due to cognitive dysfunction.

To examine the role of psychosocial stressors as risk factors in psychological illnesses (i.e. depression or anxiety), the impact of an “objectively” stressful event should be determined by one’s perceptions of their stressfulness^21^. Cohen et al. developed the perceived stress scale, which is one of the most commonly used scales to measure the degree to which situations in one’s life are appraised as stressful^20,21^. Our results revealed that post-stroke neuropsychiatric symptoms are correlated with JPSS scores, suggesting that post-stroke mood disorders are associated with mental stress. However, our results also demonstrated a weak relation between PSD and anxiety and the severity of physical impairment (paresis measured obtained using the BRS). It may not always be as simple as when the symptoms are severe, the mental stress increases, leading to the easy onset of depressed. This is because even when stress is applied, patients tend to deal with the stress to prevent depression; however, if they are vulnerable to stress due to stroke (threshold hypothesis), the introduction of a sudden and unpredictable life-threatening stressor called stroke could potentially lead to mood disorders^5,17^. Thus, the perceived stress significantly affects post-stroke neuropsychiatric symptoms over objective stress measures.

The aetiology of the post-stroke mood disorder (depression, apathy, and anxiety) is believed to be multifactorial and is poorly understood^16^. Additionally, cognitive impairment, stroke severity, and physical disability have been the most consistently identified associated factors^2,16^. In this study, we attempted to predict post-stroke mood disorders using machine learning by inputting the abovementioned factors, and obtain high prediction accuracies for cases of depression, apathy, and anxiety. Currently, a diagnostic kit for major depression is used to diagnose PSD; however, unlike major depression, PSD is characterised by variation and different pathological conditions, and hence an accurate diagnosis is infeasible^16^. PSD is therapeutically resistant in comparison with major depression^2,3^, and a more detailed diagnosis of PSD, such as depressed mood, decreased motivation, and anxiety, is beneficial for treatment^16^.

## Conclusion and limitations

In conclusion, we found that post-stroke neuropsychiatric symptoms (i.e. PSD and anxiety) may be suitably identified using LLGMN based on test scores obtained from stroke patients. Furthermore, we evaluated the index contribution to each neuropsychiatric symptom using the partial KL information measure. This study is the first step in aiming to accurately diagnose PSD using data obtained in routine practice without any special equipment.

The degree of depression, apathy, and anxiety observed in this study was relatively mild in comparison with that typically observed in patients with major depression. Moreover, patients with severe comprehension deficits who could not perform the cognitive function tests were excluded from this study. Thus, these results may not be applicable to all stroke patients. To categorise the psychiatric grouping, we used simple screening tools; however, more in-depth assessment tools are desired and will considered in the future study to improve the accuracy of the diagnosis. Moreover, we intend to conduct detailed studies using MRI images to elucidate the aetiology and improve diagnostic techniques of PSD.

## Data Availability

The datasets generated and/or analysed in the current study are available from the corresponding author upon reasonable request.
